# Complement Receptor-Mediated Phagocytosis Induces Proinflammatory Cytokine Production in Murine Macrophages

**DOI:** 10.3389/fimmu.2019.03049

**Published:** 2020-01-14

**Authors:** Durga Acharya, Xiao Rui (Lisa) Li, Rebecca Emily-Sue Heineman, Rene E. Harrison

**Affiliations:** ^1^University of Toronto Scarborough, Toronto, ON, Canada; ^2^Department of Biological Sciences, University of Toronto Scarborough, Toronto, ON, Canada

**Keywords:** macrophage, phagocytosis, cytokine, inflammation, complement, Fc receptor

## Abstract

Macrophages are professional phagocytes that are uniquely situated between the innate and adaptive arms of immunity with a high capacity for phagocytosis and proinflammatory cytokine production as well as antigen presentation. Phagocytosis is a critical process to eliminate microbes, apoptotic cells and other foreign particles and is accelerated by host-generated opsonins, such as antibodies and complement. Early phagocytosis studies established the paradigm that FcγR-mediated phagocytosis was more proinflammatory than Complement Receptor (CR)-mediated uptake in macrophages. Using qPCR, cytokine antibody arrays and ELISA, we revisited this research question in primary macrophages. Using qPCR we determined that CR-mediated phagocytosis increases levels of TNF-α, IL-1β, IL-6, and MMP-9, compared to FcγR-mediated phagocytosis and control unstimulated cells. We confirmed these findings at the protein level using cytokine antibody arrays and ELISAs. We next investigated the mechanism behind upregulated cytokine production during CR-mediated phagocytosis. IκBα protein levels were reduced after phagocytosis of both IgG- and C3bi-sRBCs indicating proteolytic degradation and implicating NF-κB activation. Inhibition of NF-κB activation impacted IL-6 production during phagocytosis in macrophages. Due to the roles of calpain in IκBα and integrin degradation, we hypothesized that CR-mediated phagocytosis may utilize calpain for proinflammatory mediator enhancement. Using qPCR and cytokine antibody array analysis, we saw significant reduction of cytokine expression during CR-mediated phagocytosis following the addition of the calpain inhibitor, PD150606, compared to untreated cells. These results suggest that the upregulation of proinflammatory mediators during CR-mediated phagocytosis is potentially dependent upon calpain-mediated activation of NF-κB.

## Introduction

Macrophages, meaning large phagocytes, are a heterogeneous population of cells that are important for maintaining homeostasis, surveillance and killing of pathogens through phagocytosis (1). Phagocytosis is an evolutionarily conserved defense mechanism by which macrophages capture and kill pathogens and remove apoptotic cells into specialized intracellular compartments. Phagocytosis is mediated by scavenger receptors, Fcγ Receptors (FcγRs), and Complement Receptors (CRs) (2). FcγRs recognize immunoglobin G (IgG) that flags target pathogens and mediates their recognition by immune cells (3, 4). In mice, the classes of Fcγ receptors includes the activating receptors FcγRI, FcγRIII, and FcγIV and the inhibitory FcγRIIB receptor (5). The intracellular domains of Fcγ receptors contain the immunoreceptor tyrosine-based activation motif (ITAM) that activates downstream signaling cascades (6, 7). The ligation of IgG-opsonized particles to FcγRs leads to receptor crosslinking which activates Src tyrosine kinase resulting in tyrosine phosphorylation of the ITAM motif (8). ITAM phosphorylation is important for the recruitment and activation of Syk (9). Activation of Src and Syk facilitates the activation of downstream Cdc42, RhoA, and Rac1 that control F-actin dynamics and contribute to the formation of pseudopods around the particle (2, 10, 11). Alternatively, the inhibitory FcγRIIB activates signaling cascades by recruiting inositol polyphosphate-5-phosphatase SHIP1 to counteract signaling from activating FcγRs (7).

The other major opsonin is the complement fragment, C3bi, which binds to targets directly or through IgM, and is recognized by CRs on phagocytes (2). The complement system contains 30 soluble and membrane bound proteins and the opsonin, C3bi, is generated from a series of biochemical reactions (12). The complement system is activated by pattern recognition receptors that have evolved to recognize antibodies, mannan-binding lectin, ficolins, C-reactive protein, C1q, and IgM (13). There are three pathways by which target molecules are opsonized by C3bi; the classical pathway, the lectin pathway and the alternative pathway. Each pathway leads to the formation of C3b/C3bi despite the differences in the initiation of the biochemical cascade. The classical pathway is activated upon the binding of C1q directly to pathogen surface or to immune complexes (12). The lectin pathway is activated by the recognition of pathogen molecular patterns by lectin receptors. Lastly, the alternative pathway is activated by the direct reaction of the C3 molecule with carbohydrate, lipid, and/or protein motifs found on the pathogen surface (14). Ultimately, each pathway generates the C3 convertase, C4bC2a for the lectin pathway, or C3bBb, for the alternative pathway. The C3 convertase leads to the production of C3bi, an opsonin (15). Receptors for the C3b protein fragments include CR1 and CRIg. While receptors for C3bi include CR2, CR3 (also known as Mac-1, CD11b/CD18, or αmβ2), and CR4 which belong to the β2 integrin family (14). CR3 requires an inside-out activation signal to effectively bind and internalize C3bi-coated particles (16). CR-mediated phagocytosis does not rely on Syk like FcγR-mediated phagocytosis but does require RhoA (17, 18). Unlike FcγR-mediated phagocytosis that involves pseudopods, CR-mediated phagocytosis leads to the formation of membrane ruffles that capture C3bi-opsonized target particles (19).

At the macroscopic level, inflammation is characterized by the presence of redness, swelling, heat, pain, and loss of tissue function (20). At the cellular level, inflammation is orchestrated with the help of histamines, cytokines, chemokines, and prostaglandins that are secreted by immune cells. A long-standing belief in the scientific community is that phagocytosis mediated by FcγRs is more potent at inducing inflammation than CR-mediated phagocytosis in macrophages. This is based on early studies in primary murine macrophages, a FcγR knockout mouse model and human monocyte-derived macrophages. The original murine macrophage study investigated whether phagocytosis induced production of arachidonic acid, a potent inflammatory mediator of vascular permeability (21). They concluded that phagocytosis via Fc receptors, but not the CR receptors, induces arachidonic acid production in murine resident peritoneal macrophages (21). Examination of human monocyte-derived macrophages revealed significant upregulation of arachidonic acid and H_2_O_2_ after uptake of IgG-opsonized, but not C3bi-opsonized particles (22). Together these studies established the paradigm that phagocytosis of IgG-opsonized targets was proinflammatory, while C3bi-opsonized targets were non-stimulatory to macrophages.

Our lab was interested in understanding how and why phagocytosis, an effector response, would enhance inflammation. We assessed levels of inflammatory cytokines produced by murine macrophages challenged with either IgG-opsonized or C3bi-opsonized particles. Interestingly, we observed significantly more proinflammatory cytokine production, at the mRNA and protein level, after CR-mediated phagocytosis vs. FcγR-mediated phagocytosis. To examine the mechanism behind proinflammatory cytokine production during phagocytosis, we examined signaling within the AP-1 and NF-κB gene expression pathways after particle engulfment. We show that CR-mediated phagocytosis activates NF-κB and provide evidence for a role for calpain in mediating proinflammatory cytokine production in macrophages. Together these studies address the current paradigm in the field and provide mechanistic insight into proinflammatory cytokine production in macrophages during phagocytosis.

## Materials and Methods

### Reagents and Antibodies

Dulbecco's modified Eagle's medium (DMEM) and fetal bovine serum (FBS) and fetal calf serum (FCS) were obtained from Wisent Inc. (Saint-Jean-Baptiste, QC). Phosphate buffered saline (PBS) was purchased from ThermoFisher Scientific Inc. (Waltham, MA). Murine macrophage-colony stimulating factor (M-CSF) and granulocyte macrophage-colony simulating factor (GM-CSF) were purchased from PeproTech Inc. (Rocky Hill, NJ). Piceatannol was from Calbiochem Novabiochem Corp. (San Diego, CA).

Sheep red blood cells (sRBCs) (10% suspension) were purchased from MP Biomedicals LLC (Irvine, CA). 3.87 μm polystyrene divinylbenzene beads (LBs) were purchased from Bangs Laboratories Inc. (Fishers, IN). Rabbit polyclonal anti-IκBα, rabbit polyclonal anti-NF-κB, rabbit polyclonal anti-p44/42 MAPK, rabbit polyclonal anti-phospho-p44/p42 MAPK antibodies, and protease/phosphatase cocktail inhibitor were all purchased from Cell Signaling Technology (Danvers, MA). Horseradish peroxidase (HRP)-conjugated secondary mouse and rabbit antibodies, AffiniPure donkey polyclonal anti-rabbit and donkey anti-human cyanine Cy^TM^ 5 and AffiniPure donkey polyclonal anti-rabbit cyanine Cy^TM^ 3 secondary antibodies were purchased from Jackson Immunoresearch Laboratories Inc. (West Grove, PA). Alexa Fluor^®^ rhodamine phalloidin was purchased from Thermo Fisher Scientific. Complement 5 (C5)-deficient human serum, Phorbol 12-Myristate 13-Acetate (PMA), Lipopolysaccharide (LPS) from *Salmonella enterica serovar typhimurium*, interferon-γ (IFN-γ), IgG and IgM opsonins, JSH-23, and Bortezomib were all purchased from Sigma Aldrich Canada Co. (Oakville, ON).

### Primary Macrophage Isolation and Cell Culture

Primary bone marrow-derived macrophages (BMDMs) were obtained and differentiated from the femur and tibia of 4–8-weeks old C57BL/6 female mice (Charles River Laboratories, Saint Constant, QC). Isolated cells were washed with PBS and resuspended in DMEM containing 10% heat-inactivated FBS, supplemented with 100 IU/mL of penicillin, 100 μg/mL streptomycin, and 20 ng/mL of M-CSF. The cells were then transferred to a 37°C incubator at 5% CO_2_ for 24 h. After the incubation, the floating cells were collected by centrifugation and the adherent cells were discarded. The cells were resuspended in DMEM containing 10% heat inactivated FBS, supplemented with 100 IU/mL of penicillin, 100 μg/mL streptomycin and 100 ng/mL of M-CSF, plated onto T75 flasks and incubated at 37°C with 5% CO_2_. The progenitor cells were then allowed to differentiate into BMDMs for 5 days. Adherent cells were removed from tissue culture flasks through gentle scraping. For experiments, 7.5 × 10^5^ cells/well were plated into 6-well dishes or 2.5 × 10^5^ cells/well were plated into 24-wells after which the BMDMs were grown to 70–90% confluency for 1 day prior to phagocytosis assays. Peritoneal macrophages were attained by lavage of the peritoneal cavity with 5 ml of ice cold 3% FCS in PBS. For each experiment, peritoneal macrophages were extracted and pooled from 2 mice. Cells were resuspended in DMEM containing 10% heat inactivated FBS and 1.5 × 10^6^ cells were plated into a 6-well and allowed to adhere for 2 h. Cells were washed twice with warmed PBS to remove non-adherent cells and serum starved for 1 h prior to PMA activation and phagocytosis. Animal studies were conducted under protocols approved by the University of Toronto Local Animal Care Committee.

### Opsonization and Phagocytosis Assays

Sheep RBCs or 3.87 μm LBs were used as target particles for macrophages in phagocytosis assays. BMDMs were incubated in serum-free DMEM at 37°C at 5% CO_2_ for 1.5 h before the phagocytosis assays. Prior to opsonization, 100 μL of sRBCs (10% suspension) were washed with 500 μL of PBS and then opsonized with 2 mg/mL of anti-sheep rabbit IgG or 0.06 mg/mL of anti-sheep rabbit IgM for 1 h on a rotator at RT. The LBs were opsonized with 2 mg/mL of human IgG or human IgM. After opsonization, IgM-opsonized sRBCs/LBs were washed with 500 μL of PBS supplemented with 0.5 mM CaCl_2_ and 0.5 mM MgCl_2_. C5-deficient human serum (50 μL) was then added to the sRBCs/LBs and incubated in a water bath at 37°C for 30 min. The C3bi/IgM-opsonized sRBCs/LBs were then washed twice with 500 μL of supplemented PBS before being used in phagocytosis assays. Forty to Sixty microliter of opsonized particles were added to macrophages to induce an average uptake of 1–2 particles per macrophages. CR was activated in macrophages with either 100 nM PMA (19) in serum-free DMEM for 7 min or 300 U/ml GM-CSF for 1 h (23, 24) prior to the addition of C3bi-sRBCs/LBs.

### mRNA Isolation and qPCR

For qPCR assays, macrophages were challenged with either C3bi- or IgG-opsonized sRBCs or LBs for 3 h. Unbound sRBCs or LBs were removed by washing with cold PBS 3 times. mRNA was extracted using the Invitrogen PureLink RNA Mini Kit (Thermo Fisher Scientific). A total of 1 μg of RNA was used for cDNA synthesis (Invitrogen SuperScript III First-Strand Synthesis SuperMix) after DNase treatment (Invitrogen DNase I amplification Grade kit). The expression levels of TNF-α, IL-6, IL-1β, and MMP-9 were measured using qPCR with the primers listed in Table 1 below. The PCR reaction was carried out with an initial denaturing step at a temperature of 95°C for 3 min, followed by 40 cycles of 30 s at 95°C (denature), 30 s at 55–62°C (annealing) and 30 s at 72°C (extension). A melt curve analysis was also conducted with each qPCR run to detect any non-specific primer binding. The housekeeping genes *ActB* or *18s* (Realtimeprimers.com; Elkins Park, PA) were used to normalize the data. iQ^TM^ SYBR Green (Bio-Rad; Mississauga, ON) was used as the detection method and the qPCR reaction was carried out with a DNA Engine Opticon System (Bio-Rad Laboratories Inc., Hercules, CA). The data was analyzed using the method of double delta Ct analysis (2^−ΔΔCt^).

**Table 1:** Murine specific qPCR primer sequences (5′-3′).

**Primer**	**Forward sequence**	**Reverse sequence**	**Annealing temperature (^**°**^C)**
TNF-α	5′-CCACATCTCCCTCCAGAAAA-3′	5′-AGGGTCTGGGCCATA GAACT-3′	58
IL-6	5′- GACAAAGCCAGAGTCCTTCAGAGAG-3′	5′-CTAGGTTTGCCGAGT GATCTC-3′	55
MMP-9	5′-CTTCTGGCGTGTGAGTTTC CA-3′	5′-ACTGCACGGTTGAA GCAAAGA-3′	62
IL-1β	5′-AGTTGACGGACCCCAAAAG-3′	5′-AGCTGGATGCTCTCA TCAGG-3′	56

### Cytokine Antibody Array and ELISAs

A mouse cytokine antibody array (Mouse Cytokine Array C1) was purchased from Ray Biotech Inc. (Peachtree Corners, GA). BMDMs were plated onto 6-well tissue culture plates and incubated at 37°C and 5% CO_2_ for 24 h. BMDMs were challenged with IgG-sRBCs or C3bi-sRBCs for 16 h. Conditioned medium was collected and the array performed according to manufacturer's instructions. The blots were imaged using a Chemidoc system from Bio-Rad. Densitometric analysis was performed with ImageJ software. The negative control spots on the array were selected as background and the positive control spot containing only biotinylated antibody was used to normalize the spot intensity (similar to a housekeeping protein). Relative changes in cytokine expression levels were determined by normalizing conditions to the control condition (BMDMs treated with PMA only).

ELISA Duosets (R&D Systems, Inc., Minneapolis, MN) were performed with the conditioned medium obtained from BMDMs 24 h after the addition of opsonized particles. For some experiments, unbound sRBCs were removed after 1 h of incubation with macrophages. For NF-κB inhibition experiments, 100 nM of JSH-23 or 10 ng/ml Bortezomib was added at the time of opsonized-particle addition. To inhibit Syk kinase, 50 μM of Piceatannol was added 30 min before phagocytosis. ELISAs were performed according to the Manufacturer's instructions to measure the protein expression of TNF-α, IL-6, and IL-1β, along with the use of the DuoSet ELISA ancillary reagent kit. Color intensity was measured utilizing the Synergy Neo2 Multi-Mode Microplate Reader (BioTek Instruments Inc., Winooski, VT). Data was analyzed through a Dunnett's multiple comparisons test to the control condition without PMA.

### Immunoblotting and Densitometry Analysis

BMDMs were challenged with either IgG- or C3bi-opsonized sRBCs for 1.5 h. Total cell lysates were extracted by scraping cells in RIPA buffer. The RIPA buffer was made by diluting 5X RIPA buffer [containing Tris-HCl (250 nM pH 7.4), NaCl (750 mM), Triton X-100 (5%), Sodium Deoxycholate (2.5% w/v), SDS (0.5%)] (Bio Basic Canada Inc., Markham, ON) with ddH2O, and 1X protease/phosphatase inhibitors [containing aprotinin (80 μM), bestatin (4 mM), E-64 (1.4 mM), leupeptin (2 mM), pepstatin A (1.5 mM) (Sigma)]. Protein concentrations were determined at 750 nm using the DC^TM^ Protein Assay kit (Bio-Rad) according to Manufacturer's instructions. Equal amounts of protein were loaded and separated using 10% SDS-PAGE. After transfer, membranes were blocked for 1 h with either 5% skim milk or 5% BSA in Tris Buffered Saline containing Tween20. Primary antibodies were diluted in blocking solutions at the following dilutions: IκBα (1/1,000), p-ERK (1/1,000), or ERK1/2 (1/1,000) and incubated with membranes overnight at 4°C. The loading control was β-actin (1/5,000). Membranes were then incubated with either rabbit or mouse HRP-coupled secondary antibodies (1/1,000) for 1 h at RT. Blots were washed 3 times for 5 min and then visualized using SuperSignal West Pico Chemiluminescent Substrate Kit (Thermo Fisher Scientific Inc.). Densitometric analysis was conducted from three independent experiments using ImageJ software. The blots used for analysis were not saturated or over-exposed.

### Immunostaining and Fluorescent Imaging

After 3 h of phagocytosis, macrophages were fixed with 4% paraformaldehyde in PBS for 20 min. Any external LBs were detected using a donkey anti-human AffiniPure Cy^TM^5 secondary antibody against human IgG. The cells were permeabilized using 0.1% Triton X-100 in PBS supplemented with 100 mM glycine for 20 min. Cells were then blocked using PBS containing 5% FBS for 1 h. Cells were incubated with NF-κB (1:200) diluted in PBS with 1% FBS for 1 h followed by labeling with a donkey anti-rabbit Cy^TM^3 secondary antibody (1:1,000) in PBS with 1% FBS for 1 h. For nuclear staining, cells were washed twice with ddH_2_O and incubated for 10 min with DAPI (1:10,000). Cells were then mounted using Dako Fluorescent Mounting Medium (Agilent Technologies Inc., Santa Clara, CA). Cells were visualized using a 63x oil-immersion objective using an inverted Zeiss epifluorescent microscope equipped with AxioVision software (Carl Zeiss Canada Ltd., North York, ON). Linear adjustments (contrast and brightness) to images acquired by epifluorescence microscopy were conducted using Axiovision software. Non-linear adjustments were not made. Figures were prepared using Adobe Illustrator CS6 (San Jose, CA).

### Calpain Inhibitor Assay

BMDMs were challenged with opsonized sRBCs as described above for 1 h. Then 100 μM of calpain inhibitor diluted in DMSO (PD150606, Sigma Aldrich) was added to each condition for 1 h. mRNA was isolated followed by qPCR for cytokine expression analysis, as described above. For the cytokine antibody array, phagocytosis was performed for 8 h, with 100 μM of PD150606 added 1 h after phagocytosis. After 8 h, the conditioned supernatant was collected, and the cytokine array assay performed as described earlier.

### Data Analysis and Statistics

The statistical analysis was conducted on Prism from GraphPad Software Inc. (La Jolla, CA). A two-way ANOVA was used followed by Tukey's or Dunnett's test for multiple comparisons. Differences with a confidence interval of 95% between the specified conditions and the control (generally the PMA-stimulated macrophages unless otherwise indicated) was considered significant during analysis of the data. The data is represented as the mean ± standard error of the mean (S.E.M.) from three biological replicates.

## Results

### CR-Mediated Phagocytosis Is Proinflammatory

**Figure 1:**
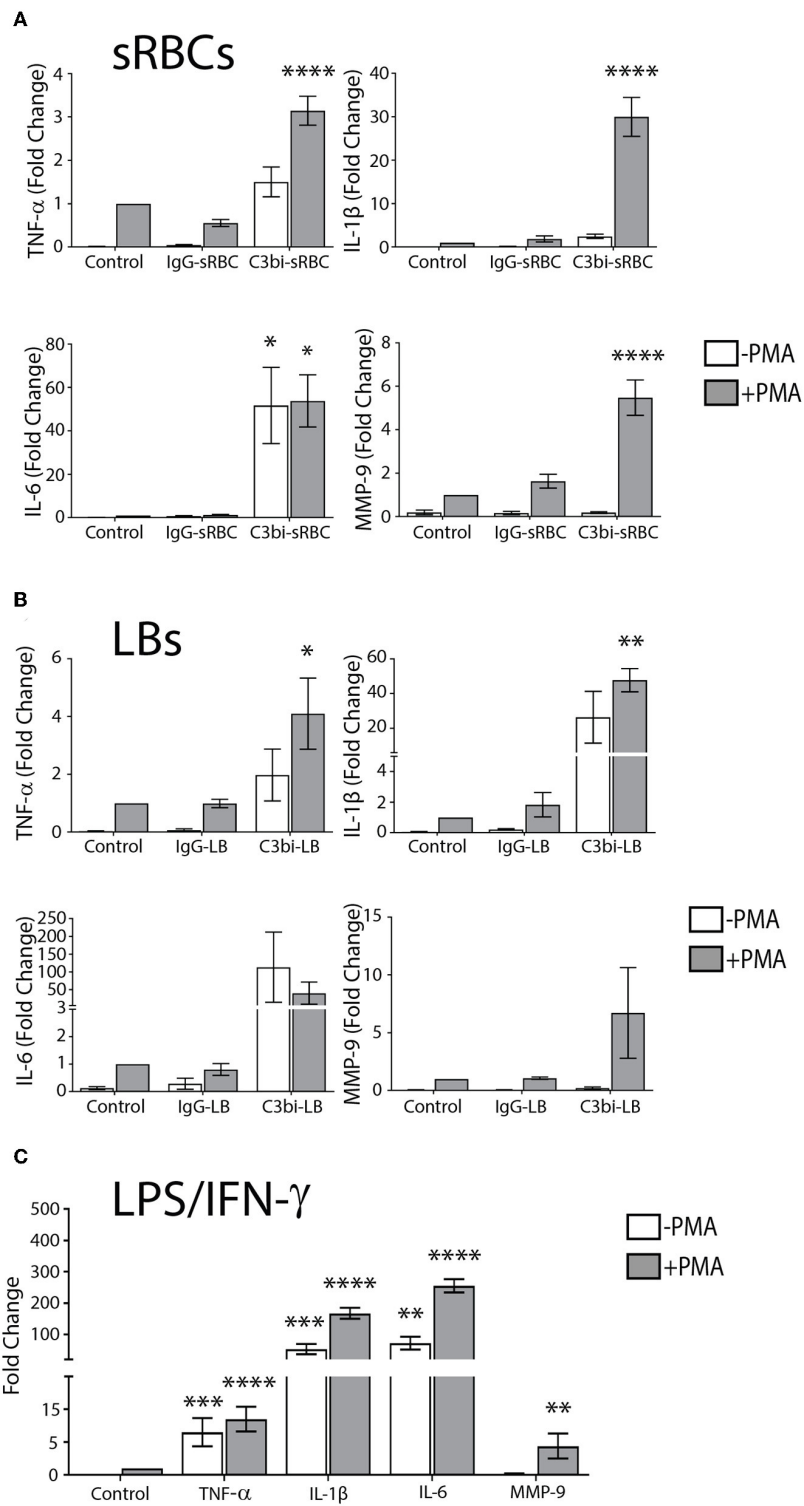
CR-mediated phagocytosis promotes higher expression of proinflammatory molecules compared to FcγR-mediated phagocytosis. **(A)** Expression levels of mRNA of TNF-α, IL-1β, IL-6, and MMP-9 in control BMDMs or BMDMs after 3 h of ingestion of IgG-sRBCs or C3bi-sRBCs. **(B)** Fold change gene expression of TNF-α, IL-1β, IL-6, and MMP-9 after 3 h of phagocytosis of opsonized latex beads (LBs) in BMDMs. **(C)** Gene expression changes in BMDMs stimulated with LPS (50 ng/mL) and IFN-γ (100 ng/mL) for 3 h, compared to resting cells. Expression levels were normalized using β-actin and the fold change was calculated relative to the control condition with PMA. A two-way ANOVA followed by Tukey's multiple comparison was performed and significance of each condition was evaluated relative to PMA-stimulated control cells (^*^p < 0.05, ^**^p < 0.01, ^***^p < 0.001, ^****^p < 0.0001). Data are plotted as the mean ± S.E.M. from three independent experiments.

It is widely believed that FcγR- but not CR-mediated phagocytosis is proinflammatory in macrophages (21, 22, 25). However, the mechanism by which FcγR signals to transcription factors (e.g., NF-κB) has not been elucidated. We revisited the early biochemical studies of phagocytosis in murine macrophages and used qPCR and sensitive, quantitative protein assays to examine the levels of proinflammatory mediators. To begin, we investigated mRNA levels of TNF-α, IL-1β, IL-6, and MMP-9 in bone marrow-derived murine macrophages (BMDMs) after phagocytosis of IgG- or C3bi-opsonized targets. These inflammatory proteins play an important role in the initiation, progression and resolution of inflammation and thus were chosen as markers of inflammation (26). PMA was used to activate the macrophage complement receptors, α_M_β_2_, to enable uptake of C3bi-opsonized sRBCs (27). However, PMA can signal downstream to activate NF-κB (28) so we used BMDMs only stimulated with PMA as the negative control, to which the other conditions were normalized to. Phagocytosis assays were carried out in serum-free DMEM to avoid non-specific activation of receptors by serum components (29). The qPCR data presented in Figure 1A shows the transcript levels of TNF-α, IL-1β, IL-6, and MMP-9 in control cells and BMDMs after ingesting either IgG-sRBCs or C3bi-sRBCs for 3 h. PMA-stimulated BMDMs challenged with C3bi-sRBCs produced significantly higher levels of TNF-α, IL-1β, IL-6, and MMP-9 compared to control BMDMs. Significant upregulation of these proinflammatory genes was not observed in BMDMs that had internalized IgG-sRBCs. Unexpectedly, resting BMDMs challenged with C3bi-sRBCs produced similar levels of IL-6 compared to PMA-stimulated BMDMs challenged with C3bi-sRBCs (Figure 1A). Immunofluorescence inspection of these cells revealed a basal level of phagocytosis of C3bi-sRBCs even in the absence of PMA (data not shown). To ensure that the proinflammatory effects we observed were not due to components on the sRBC membranes, we repeated these experiments using opsonized latex beads (LBs) (Figure 1B). Very similar results were observed with opsonized LBs, compared to opsonized sRBCs (Figure 1A), with ingestion of complement opsonized LBs invoking higher expression of proinflammatory mediators vs. IgG-oponized latex beads (Figure 1B). Classically activated BMDMs stimulated with LPS/IFN-γ were used as a positive control (30) (Figure 1C).

### CR-Mediated Phagocytosis Induces More Cytokine Secretion Than FcγR-Mediated Phagocytosis

**Figure 2:**
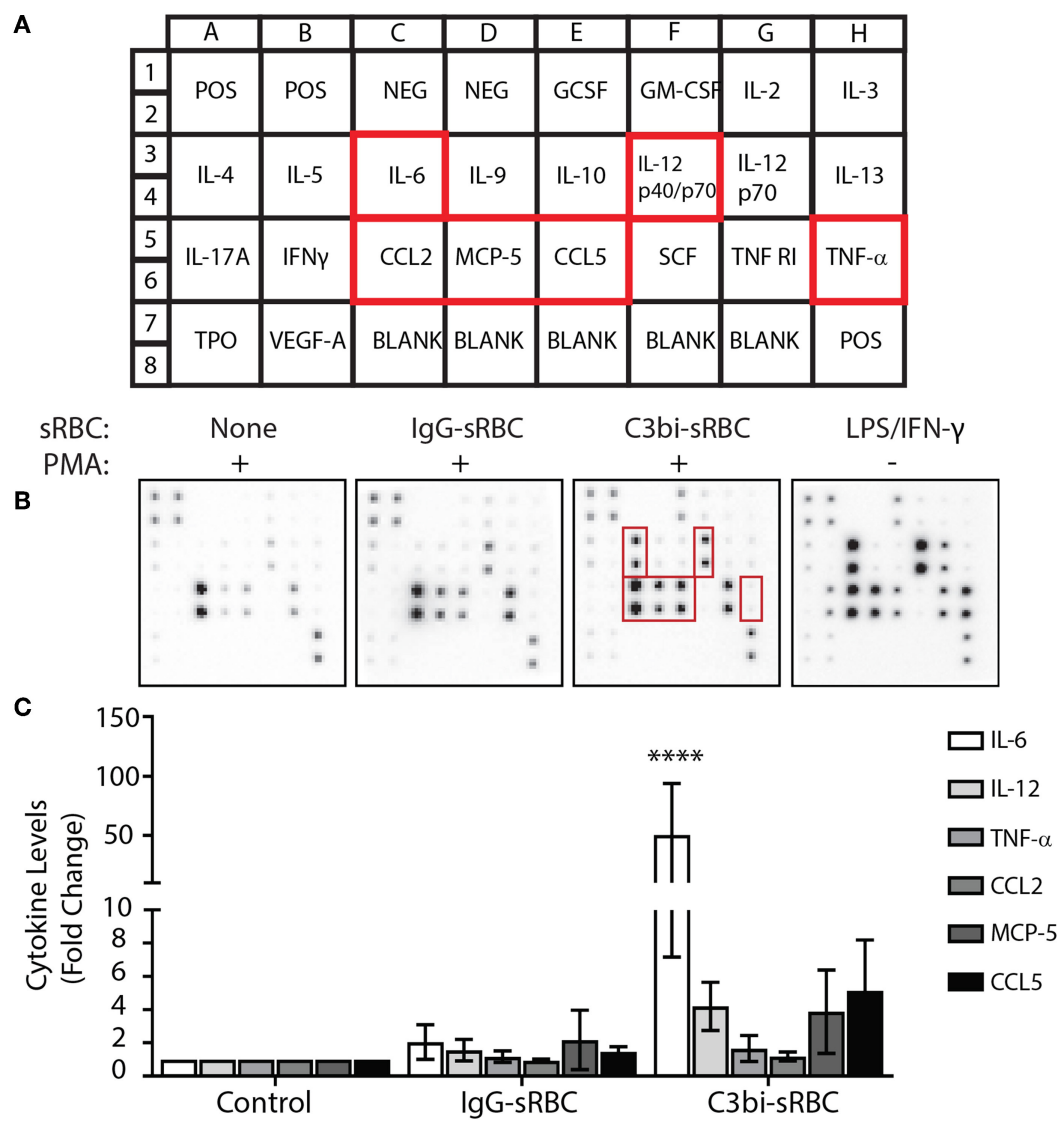
CR-mediated phagocytosis leads to higher secreted proinflammatory proteins than FcγR-mediated phagocytosis. **(A)** Schematic of cytokines and chemokine antibodies present on the cytokine antibody array from Ray BioTech. **(B)** Representative images of the cytokine array membrane after exposing conditioned supernatants from the following experimental conditions; no phagocytosis + PMA, IgG-sRBCs + PMA, C3bi-sRBCs + PMA, and LPS/IFN-γ treatment for 16 h. **(C)** Densitometry analysis for IL-6, IL-12, TNF-α, CCL2, MCP-5, and CCL5 of replicate array blots. Expression level was normalized to positive biotinylated antibody signal spots and then to the no phagocytosis + PMA condition for each cytokine. A two-way ANOVA followed by Dunnett's multiple comparison was performed. The significance of each condition was evaluated relative to PMA-stimulated control cells (^****^p < 0.0001). Data are plotted as the mean ± S.E.M. from three independent experiments.

We next extended and validated our mRNA data with protein analysis of proinflammatory mediators. We first employed a cytokine antibody array to analyze the levels of secreted cytokines (Figure 2A). BMDMs were stimulated with PMA prior to phagocytosis which was allowed to proceed for 16 h to accumulate detectable cytokines within the media. The conditioned media was collected and analyzed using the cytokine array kit (Figure 2B). As a positive control, PMA-stimulated BMDMs were also treated with LPS/IFN-γ for 16 h (Figure 2B). Densitometric analysis of three replicate experiments showed enhanced proinflammatory mediator secretion following phagocytosis of C3bi-sRBCs with significantly higher IL-6 secretion compared to control cells and BMDMs that had ingested IgG-sRBCs (Figure 2C).

**Figure 3:**
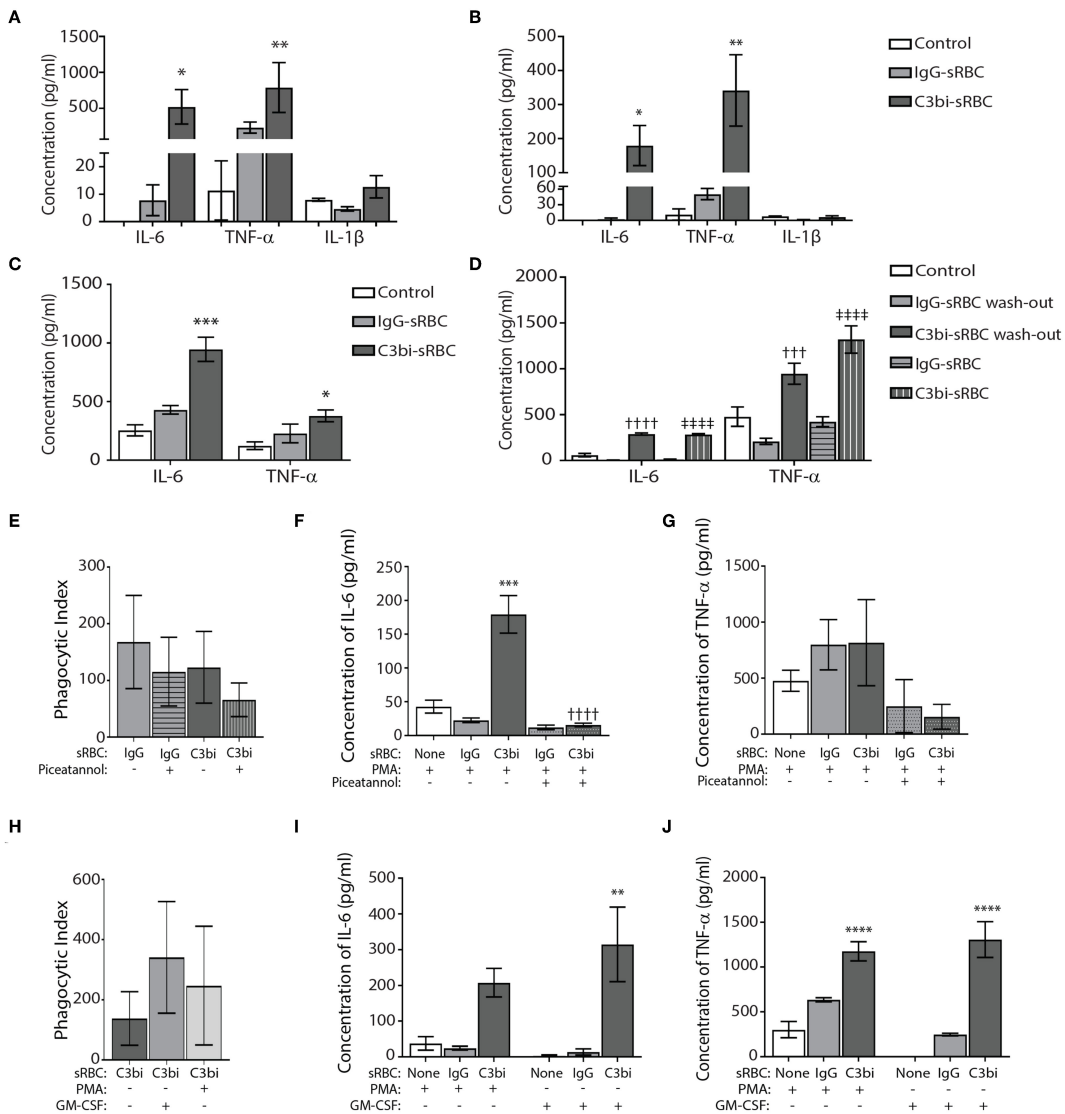
Conditions of proinflammatory cytokine production during CR-mediated phagocytosis in macrophages. **(A)** Quantification of ELISA results for TNF-α, IL-6, and IL-1β from conditioned media of BMDMs after 24 h of phagocytosis. Control cells were cells treated with PMA alone. A two-way ANOVA followed by Dunnett's multiple comparison was performed. The significance of each condition was evaluated relative to PMA-stimulated control cells (^*^p < 0.05, ^**^p < 0.01). Data are plotted as the mean ± S.E.M. from three independent experiments. **(B)** Normalization of ELISA results for TNF-α, IL-6, and IL-1β from conditioned media of BMDMs after phagocytosis to the phagocytic index of each opsonized target. A two-way ANOVA followed by Dunnett's multiple comparison was performed. The significance of each condition was evaluated relative to PMA-stimulated control cells (^**^p < 0.005, ^*^p < 0.05). Data shows mean ± S.E.M. from three independent experiments. **(C)** Peritoneal macrophages challenged with C3bi-opsonized sRBCs produce more pro-inflammatory cytokines than IgG-opsonized sRBCs. ELISA data representing secreted IL-6 and TNF-α by peritoneal macrophages primed with PMA followed by phagocytosis of C3bi- or IgG-sRBCs. Data shows mean ± S.E.M. from three independent experiments (^***^p < 0.001, ^*^p < 0.05 relative to control cells). A one-way ANOVA followed by Tukey's multiple comparison was performed. **(D)** Quantification of secreted IL-6 and TNF-α through ELISA with or without washout of opsonized-sRBCs after 1 h. Supernatant was removed after 1 h containing sRBCs and replaced with new serum free DMEM and supernatant was collected after 23 h. sRBC treatments without wash out refers to sRBCs persisting in media for the full 24 h until collection. Data are plotted as the mean ± S.E.M. from 3 independent experiments (^†††^*p* < 0.001, ^††††^*p* < 0.0001 relative to IgG-sRBC wash out), (^‡‡‡‡^*p* < 0.0001 relative to IgG-sRBC). A one-way ANOVA followed by Tukey's multiple comparison was performed. **(E)** The effects of Piceatannol treatment on the phagocytic index in BMDMs (number of ingested particles/ 100 macrophages). Immunofluorescence images were taken, and the phagocytic index was calculated as number of sRBCs per 100 macrophages. Data are plotted as the mean ± S.E.M. from three independent experiments. **(F)** The effects of Piceatannol treatment on secreted IL-6 levels and **(G)** TNF-α levels after phagocytosis for 24 h, detected using ELISA. Data is plotted as the mean ± S.E.M. from three replicate trials (^***^p < 0.001 relative to control) (^††††^*p* < 0.0001 relative to C3bi without Piceatannol). A one-way ANOVA followed by Tukey's multiple comparison was performed. **(H)** Stimulation of BMDMs with GM-CSF (300 U/ml) for 1 h increases phagocytosis of C3bi-sRBCs. Phagocytosis was allowed to proceed for 1 h and then cells were fixed and imaged. The phagocytic index was calculated as number of sRBCs per 100 macrophages. Data plotted as the mean ± S.E.M. from three independent experiments **(I)** ELISA data representing secreted cytokine levels of IL-6 and **(J)** TNF-α from BMDMs challenged with IgG- or C3bi-sRBCs after either: no treatment, 7 min PMA pretreatment, or 1 h pretreatment with GM-CSF. Data plotted as the mean ± S.E.M. from three independent experiments (^**^p < 0.01 relative to no sRBC with GM-CSF) (^****^p < 0.0001 relative to no sRBC with or without GM-CSF). A one-way ANOVA followed by Tukey's multiple comparison was performed.

We extended the time period of phagocytosis to 24 h and utilized ELISAs to examine select proinflammatory cytokines. Significantly more TNF-α and IL-6 was secreted from BMDMs after ingestion of C3bi-sRBCs, compared to cells undergoing phagocytosis of IgG-sRBCs (Figure 3A). This trend was consistent when the cytokine levels detected by ELISA were normalized to the phagocytic index (number of ingested particle/100 macrophages) for each opsonized target (Figure 3B). To see if this was limited to bone marrow-derived macrophages, we also investigated proinflammatory cytokine production during phagocytosis in mouse peritoneal primary macrophages. Conditioned media after 24 h of phagocytosis in mouse peritoneal macrophages was subjected to ELISA and both IL-6 and TNF-α levels were significantly increased after ingestion of C3bi-sRBCs, compared to IgG-sRBCs, or conditioned media from control peritoneal macrophages (Figure 3C). Typically for our assays, we expose macrophages to an excess of opsonized targets over the experimental time period. To ensure that unbound sRBCs were not dying/ degrading and inducing an inflammatory reaction in macrophages, we washed off unbound sRBCs after 1 h of phagocytosis and compared cytokine levels to macrophages exposed to a constant supply of sRBCs. Levels of IL-6 and TNF-α in conditioned media was not significantly different in the sRBC “wash-out” experiments compared to cytokine levels in BMDMs continuously exposed to opsonized targets (Figure 3D). We were next interested in whether particle internalization itself induced proinflammatory cytokine production or whether particle internalization induced the inflammatory cascade. We pretreated macrophages with 50 μM Piceatannol for 30 min to inhibit Syk kinase (31, 32) prior to phagocytosis assays. We monitored phagocytosis and while there were less ingested IgG-sRBCs and C3bi-sRBCs compared to untreated controls, the results were not significant (Figure 3E). At 24 h cytokine levels were measured and a significant reduction in IL-6 was observed in Piceatannol-treated macrophages ingesting C3bi-sRBCs, compared to untreated control cells (Figure 3F). TNF-α levels were also reduced after CR-mediated phagocytosis compared to untreated BMDMs ingesting C3bi-sRBCs but these results were not significant (Figure 3G). Based on the continued uptake of sRBCs in the presence of Piceatannol (Figure 3E) the effects of particle uptake inhibition on proinflammatory cytokine production remain inconclusive. Finally, we wanted to assess whether PMA was amplifying the proinflammatory cytokine expression in macrophages. To address this we activated integrins in BMDMs using 300 U/ml GM-CSF (23, 24). We first confirmed that treatment of macrophages with GM-CSF enhanced phagocytosis (Figure 3H). We next performed phagocytosis for 24 h in GM-CSF-stimulated macrophages and collected conditioned media to analyze by ELISA. Both IL-6 (Figure 3I) and TNF-α (Figure 3J) were significantly upregulated in BMDMs ingesting C3bi-sRBCs after GM-CSF stimulation compared to macrophages not undergoing phagocytosis.

### p-ERK Levels Remain Constant, but IκBα Protein Levels Are Reduced After Ingestion of C3bi- or IgG-sRBCs in BMDMs

**Figure 4:**
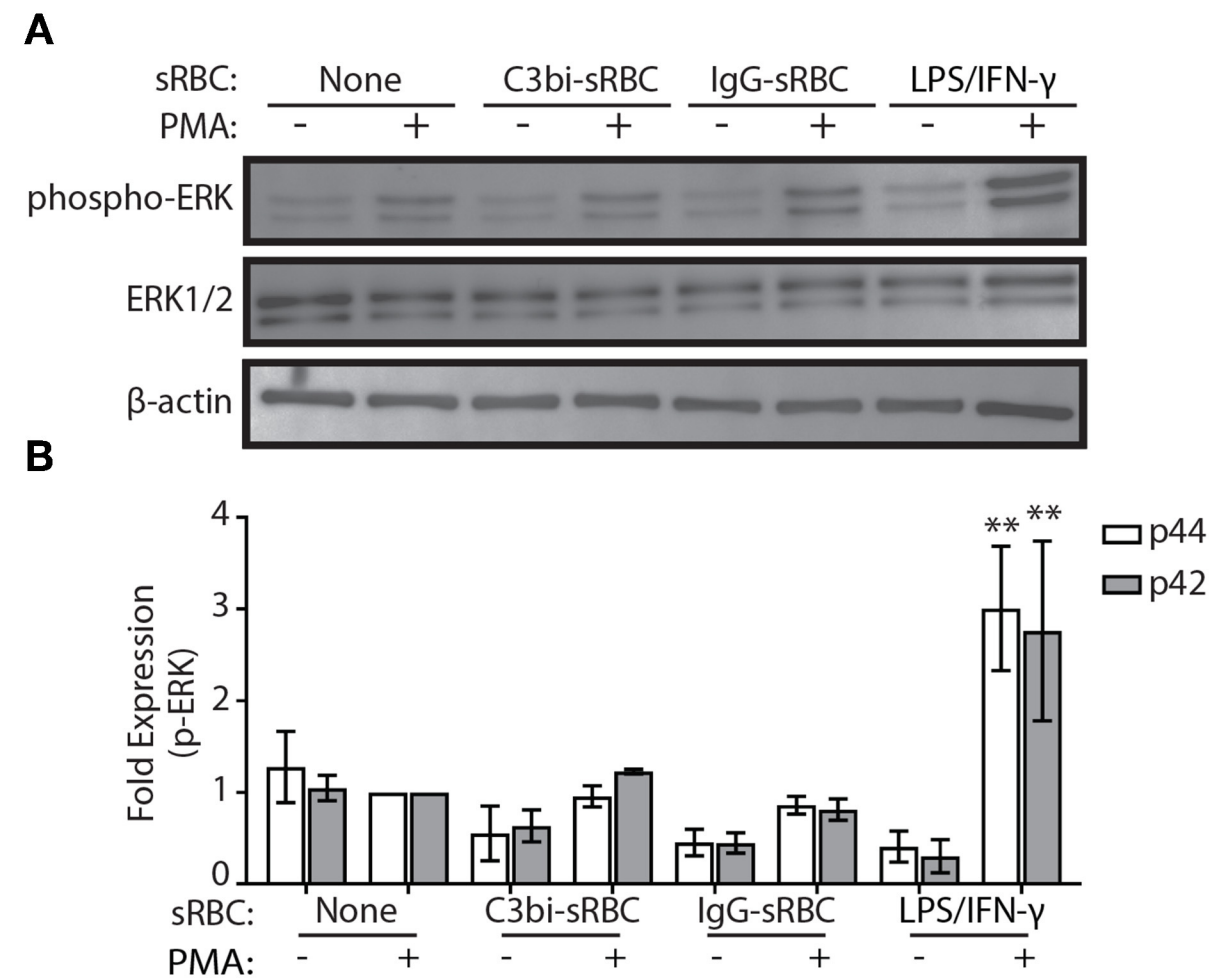
ERK is not activated during phagocytosis of C3bi- or IgG-opsonized particles in BMDMs. **(A)** Representative immunoblots showing phosphorylated ERK and total ERK in BMDMs challenged with C3bi- or IgG-opsonized sRBCs for 1.5 h. BMDMs treated with LPS/IFN-γ for 1.5 h was used as a positive control. β-actin was used as a loading control. **(B)** Densitometric analysis of p-ERK (p44/p42) levels normalized to β-actin. Data are plotted as the mean ± S.E.M. from three independent experiments. Significance relative to the control cells with PMA was determined using two-way ANOVA followed by Tukey's multiple comparison (^**^p < 0.01).

Gene upregulation of many proinflammatory mediators is mediated by NF-κB or AP-1 (33). Previous work on CR3 has shown that co-activation with dectin receptors induces TNF-α and IL-6 secretion via the Syk-JNK-AP-1 signaling pathway (34). Since ERK is an upstream element of AP-1 activation (35), we first investigated the phosphorylation status of ERK1/2 using immunoblotting. Since we observed gene expression changes after 3 h of phagocytosis, we investigated earlier time points for ERK activity. Phagocytosis was performed in BMDMs for 1.5 h and lysates collected for immunoblotting (Figure 4A). Densitometry analysis of immunoblots from three biological replicates indicated that there was no significant difference in the level of p-ERK in either C3bi-sRBC-challenged BMDMs or BMDMS ingesting IgG-sRBCs (Figure 4B). Stimulation of BMDMs with LPS/IFN-γ did cause enhanced levels of phosphorylated ERK1/2 (Figure 4B), as expected (36).

**Figure 5:**
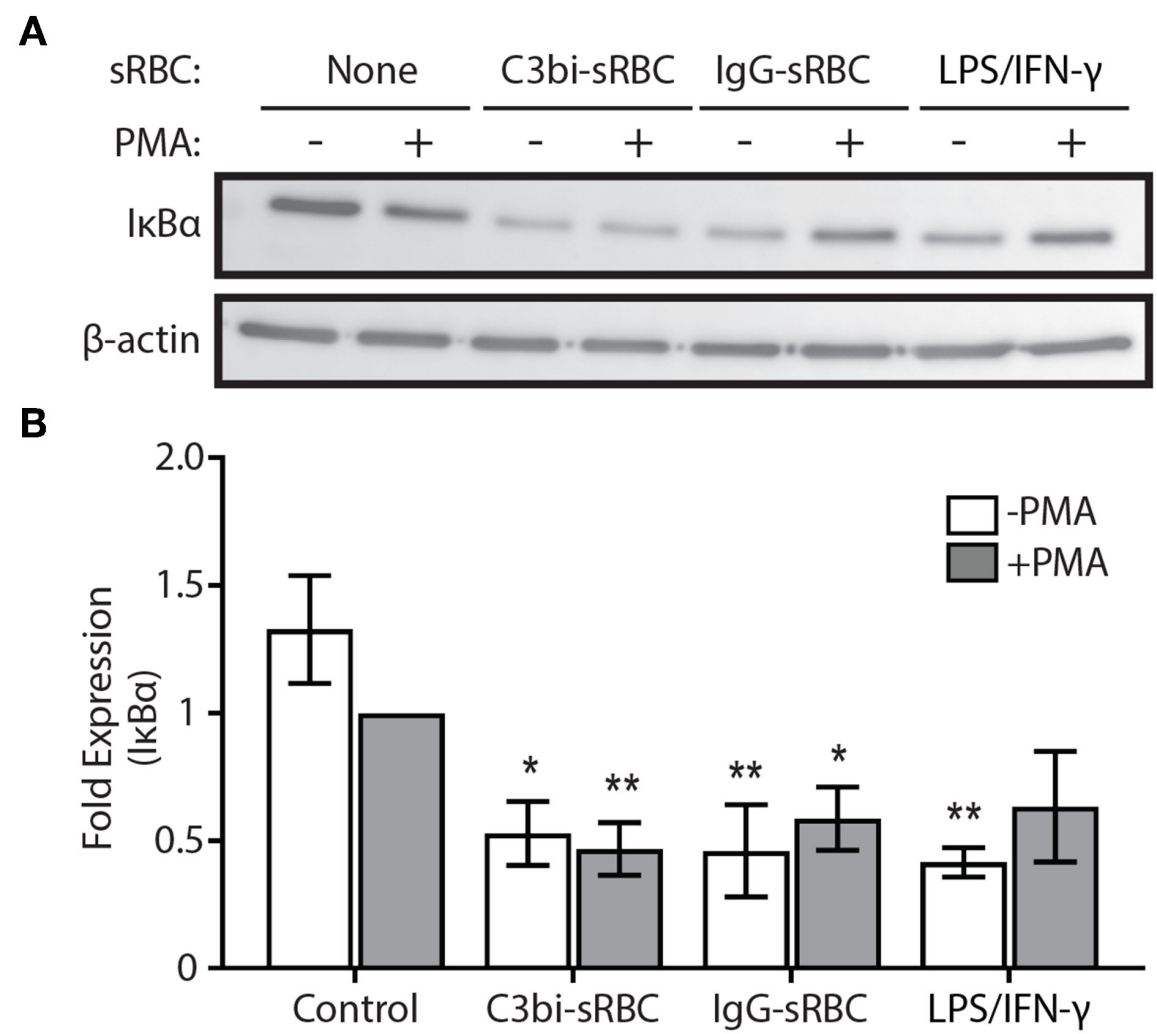
Activation of phagocytic receptors initiates IκBα degradation. **(A)** Representative immunoblot showing total IκBα protein levels in BMDMs challenged with either C3bi-opsonized sRBCs or IgG-sRBCs for 1.5 h. BMDMs were also treated with LPS/IFN-γ alone for 1.5 h as a positive control. β-actin was used as a loading control. **(B)** Densitometry analysis of 3 independent experiments. Expression levels were normalized to β-actin and then to the PMA-stimulated negative control cells. Data are plotted as the mean ± S.E.M. Significance relative to the control cells with PMA was determined using two-way ANOVA followed by Tukey's multiple comparison (^*^p < 0.05, ^**^p < 0.01).

We next turned our investigation toward the NF-κB signaling pathway and examined the protein levels of IκBα using immunoblotting. Proteosome degradation of IκBα is a critical signaling event to release of cytosol-sequestered NF-κB, allowing it to move into the nucleus for gene activation (37). The phagocytosis experiments were repeated for 1.5 h and lysates collected and probed for IκBα (Figure 5A). TLR-activation is known to induce NF-κB nuclear translocation (38) and we saw an expected reduction in IκBα levels after treatment of BMDMs with LPS/IFN-γ (Figure 5B). BMDMs ingesting both C3bi- and IgG-sRBCs showed significantly lower levels of IκBα compared to control cells. These results encouraged us to look further into the involvement of the NF-κB signaling pathway in proinflammatory cytokine expression during phagocytosis in macrophages.

### BMDMs That Ingest C3bi-LBs Have Less Cytosolic NF-κB Than BMDMs That Internalize IgG-LBs

**Figure 6:**
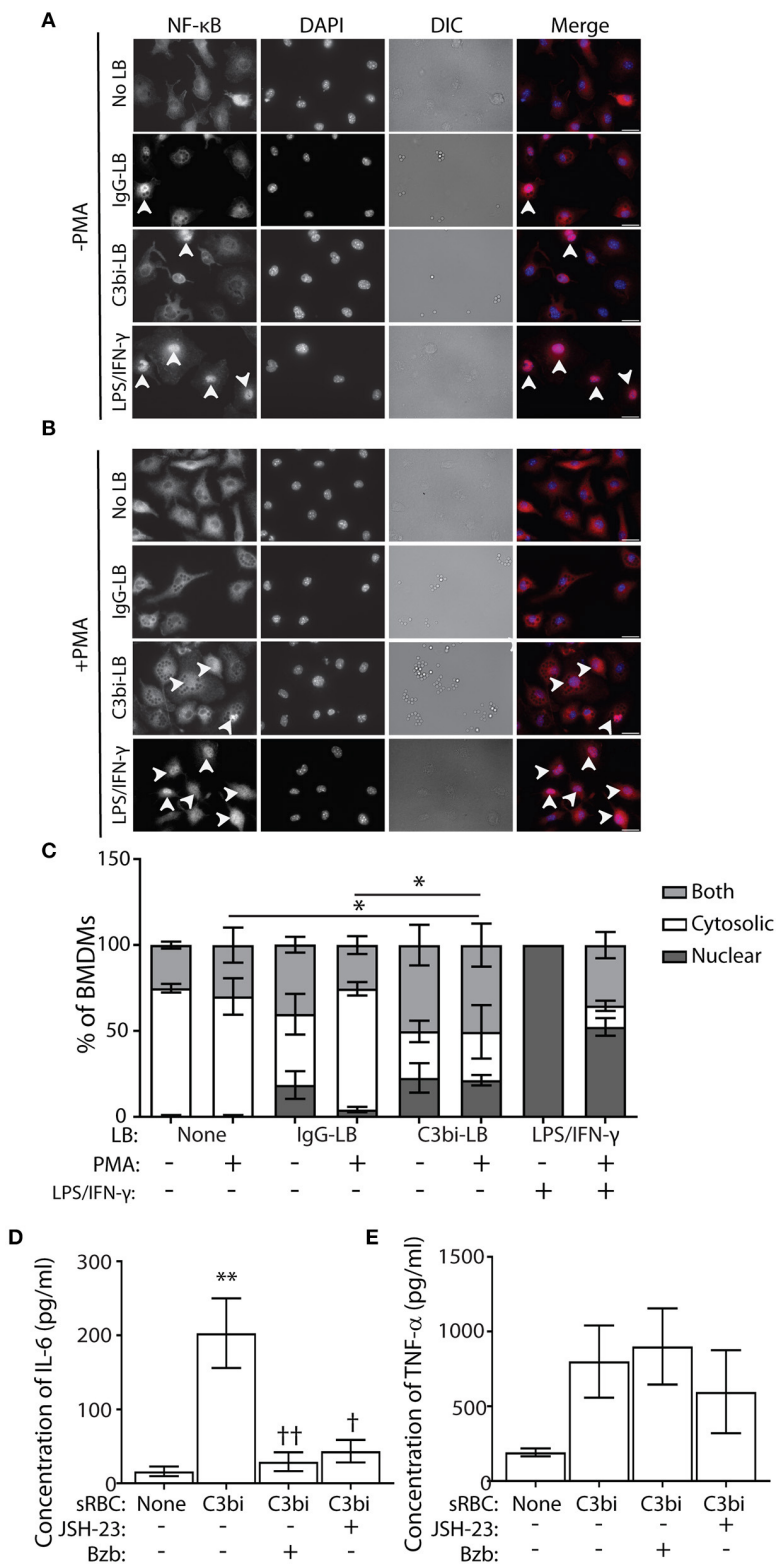
BMDMs challenged with C3bi-LBs have less cytosolic NF-κB than BMDMs challenged with IgG-LBs and NF-κB activity is required for CR-induced proinflammatory cytokine production. **(A)** Representative immunofluorescent images of untreated or **(B)** PMA-treated BMDMs after ingestion of IgG-LBs, C3bi-LBs, or exposed to LPS/IFN-γ for 3 h. Arrowheads indicate BMDMs with nuclear NF-κB. Scale bars = 20 μm. **(C)** Quantification of NF-κB subcellular distribution in BMDMs after phagocytosis or treatment with LPS/IFN-γ, with and without PMA stimulation. Cells were characterized as either having solely nuclear or cytosolic NF-κB immunostaining, or both in which strong signal for NF-κB was observed in the cytosol and the nucleus. *n* > 100 BMDMs. A two-way ANOVA followed by Tukey's multiple comparison was performed. The cytosolic localization of PMA-stimulated BMDMs challenged with C3bi-LBs was compared with PMA-stimulated BMDMs and BMDMs ingesting IgG-LBs (^*^p < 0.05). Significantly more LPS/IFN-γ-stimulated BMDMs showed nuclear NF-κB and less cytosolic staining, which is not indicated on graph. Data are plotted as the mean ± S.E.M. from three independent experiments. **(D)** Quantification of ELISA detection of secreted IL-6 and **(E)** TNF-α after treatment of BMDMS with the NF-kB inhibitors JSH-23 and Bortezomib during CR-mediated phagocytosis. Data is plotted as the mean ± S.E.M. from three independent experiments (^**^p < 0.01 relative to control condition) (^††^*p* < 0.01, ^†^*p* < 0.01 relative to C3bi-sRBC). A one-way ANOVA followed by Tukey's multiple comparison was performed.

Using immunofluorescence, we next directly examined the subcellular distribution of NF-κB, as a proxy to its gene expression activity (37). Phagocytosis was performed for 3 h with opsonized LBs for compatibility with the NF-κB antibody. Control cells were stimulated with LPS/IFN-γ for an equivalent time-period. Immunostaining revealed 3 distinct phenotypes of NF-κB subcellular distribution (Figure 6A). NF-κB immunostaining was either distinctly nuclear (with empty cytosol), or cytosolic (with empty nucleus) or “both,” for cells with NF-κB strongly labeling throughout the cell (Figure 6A). For each condition, we quantified the subcellular distribution of NF-κB from at least 100 BMDMs (Figure 6C). As expected, the positive control cells activated with LPS/IFN-γ showed predominantly nuclear NF-κB, which interestingly, was reduced with PMA treatment (Figures 6B,C). While there was no significant difference in the number of cells containing nuclear NF-κB localization after phagocytosis of either opsonized particles, compared to control cells, there was a difference in the number of cells exhibiting purely cytosolic NF-κB. After internalization of C3bi-LBs, significantly fewer BMDMs treated with PMA had cytosolic distributions of NF-κB compared to PMA-treated control cells or those ingesting IgG-LBs (Figure 6C). To more directly test the involvement of NF-κB in phagocytosis-induced proinflammatory cytokine production, we employed NF-κB activation inhibitors. We utilized a 4-Methyl-N1-(3-phenyl-propyl)-benzene-1,2-diamine (JSH-23) and Bortezomib (Bzb) to inhibit NF-kB activity (39, 40). JSH-23 is a novel aromatic compound that inhibits NF-kB activity in RAW 264.7 macrophages by preventing nuclear translocation of NF-kB without affecting IkBa degradation (39). Bzb is a reversible 26S proteasome inhibitor (40). During CR-mediated phagocytosis, we inhibited NF-kB using either 100 nM of JSH-23 or 10 ng/ml BzB and measured IL-6 and TNF-α levels after 24 h using ELISA. BMDMs challenged with C3bi-sRBCs in the presence of Bzb or JSH-23 produced significantly less IL-6 than untreated BMDMs (Figure 6D). The TNF-α levels secreted by BMDMs in the presence of the NF-kB inhibitors also showed lower levels compared to untreated macrophages ingesting C3bi-sRBCs but these results were not significant (Figure 6E).

### Calpain Inhibition Reduces Phagocytosis-Induced Upregulation of Inflammatory Cytokines

**Figure 7:**
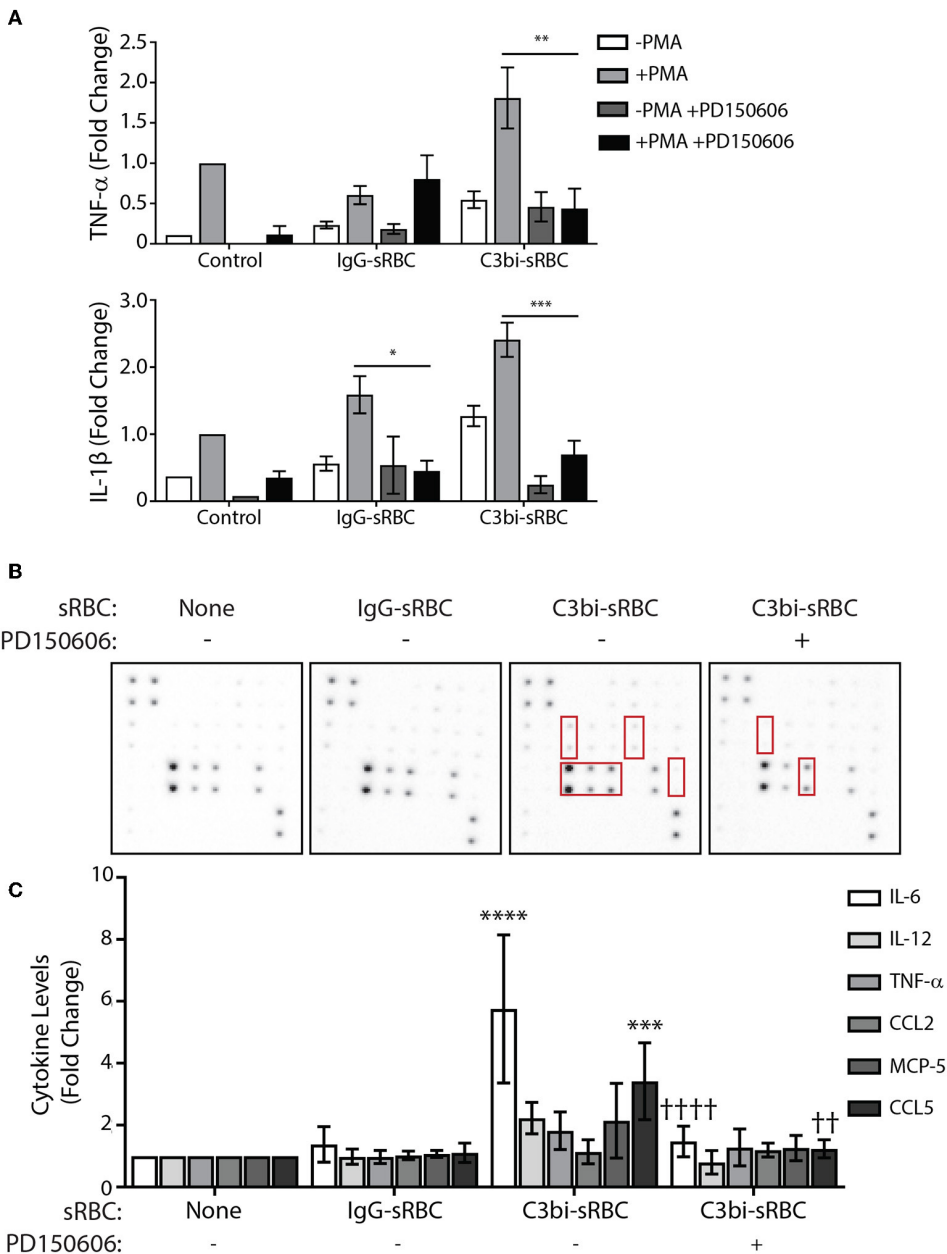
Calpain inhibition blocks proinflammatory mediator upregulation during phagocytosis in BMDMs. **(A)** qPCR data representing mRNA expression levels of TNF-α and IL-1β in BMDMs after 2 h of phagocytosis with 100 μM of PD150606 added in the last hour. Expression levels were normalized to 18S and the fold change was calculated relative to the control condition with PMA. A two-way ANOVA followed by Tukey's multiple comparison was performed (^*^p < 0.05, ^**^p < 0.01, ^***^p < 0.001). Data are plotted as the mean ± S.E.M. from three independent experiments. **(B)** Cytokine antibody array probed with conditioned media from PMA-stimulated BMDMs after 8 h of phagocytosis, with 100 μM of PD150606 included during CR-mediated phagocytosis. **(C)** Densitometry analysis of IL-6, IL-12, TNF-α, CCL2, MCP-5, and CCL5 from replicate array blots. Expression level was normalized to positive biotinylated antibody signal spots and then to the control cells + PMA condition for each cytokine. A two-way ANOVA followed by Tukey's multiple comparison was performed (^***^p < 0.001, ^****^p < 0.0001 relative to control condition), (^††^*p* < 0.01, ^††††^*p* < 0.0001 relative to untreated C3bi-sRBC). Data are plotted as the mean ± S.E.M. from four biological replicates.

To gain insight into the mechanism by which phagocytosis promotes proinflammatory cytokine and chemokine production, we turned our attention to calpain. We became interested in this cysteine protease since CR3 is an integrin and calpain is involved in integrin signaling through degradation of talin (41). Importantly, IκBα is another substrate of calpain (42), implicating a potential involvement in phagocytosis-induced cytokine production. Calpain has been shown to indirectly play a role in FcγR-mediated phagocytosis as the cleavage of ASAP2 by calpain reduced particle uptake during FcγR-mediated phagocytosis (43). We employed the calpain inhibitor, the PD150606 peptide, which inhibits the protease core domains of both calpain-1 and calpain-2 (44) and examined gene expression and cytokine and chemokine protein expression during phagocytosis in BMDMs. To avoid particle internalization issues in the presence of the calpain inhibitor, we allowed phagocytosis to occur for 1 h prior to addition of 100 μM PD150606. For qPCR studies, we continued phagocytosis for an additional hour in the presence of the peptide prior to cell extraction. We investigated TNF-α and IL-1β as they had the most robust expression after 3 h of phagocytosis of C3bi-sRBCs (Figure 1A). In untreated cells, after 2 h of phagocytosis of C3bi-sRBCs, we again saw upregulation of TNF-α and IL-1β as well as upregulation of IL-1β following IgG-sRBC ingestion (Figure 7A). Cells treated with 100 μM PD150606 had significant less IL-1β levels for both phagocytic targets, compared to untreated cells (Figure 7A). The CR-mediated upregulation of TNF-α was also significantly blunted by PD150606 treatment (Figure 7A).

We next investigated how calpain inhibition would affect the expression of cytokines at a secreted protein level during phagocytosis. Due to the instability of the peptide, the assays were only carried out for 8 h after which conditioned media was exposed to a cytokine antibody array. In control cells, significant levels of IL-6 and CCL5, a chemokine which recruit monocytes, NK and T cells to sites of inflammation (26), were detected following uptake of C3bi-sRBCs which was not observed in control cells or after ingestion of IgG-sRBCs (Figures 7B,C). However, the secreted protein levels of IL-6 and CCL5, were significantly lower from BMDMs treated with PD150606 to inhibit calpain, compared to untreated BMDMs ingesting C3bi-sRBCs (Figures 7B,C). Together, these results implicate calpain involvement in proinflammatory cytokine and chemokine secretion induced by CR-mediated phagocytosis.

## Discussion

A long-standing belief in the scientific community is that phagocytosis mediated by FcγRs is potent at inducing inflammation whereas CR-mediated phagocytosis in macrophages is non-inflammatory. Recent efforts have concentrated on understanding CR-mediated phagocytosis given the prompt, pivotal role of complement in clearing infections. Low levels of bacteria can be quickly resolved by circulating complement, while rapidly proliferating pathogens necessitate time consuming antibody-mediated solutions of the adaptive immunity (45). Studies so far have revealed striking differences between the modes of phagocytosis induced by the opsonic receptors in macrophages, including a strong requirement for the RhoA GTPase, and not rac1 and cdc42, for CR-mediated phagocytosis (46).

We revisited the inflammatory capacity of opsonic receptor signaling using quantitative mRNA and protein approaches. Surprisingly, we observed enhanced proinflammatory gene and secreted protein levels during CR-mediated phagocytosis. This deviates from previous work in mouse macrophages (21) which may be explained in part from experimental discrepancies including differences in macrophage subtype and proinflammatory readout. The original study by Aderem et al. (21) examined murine resident peritoneal macrophages and detected levels of arachidonic acid, which is a signaling fatty acid that can become inflammatory if generated by phospholipase A2 (47). Arachidonic acid is metabolized to make prostaglandins and leukotriene B4, that cause arterial dilation to enhance immune cell infiltration into the sites of inflammation (47). While arachidonic acid levels were not increased following CR activation in the original study, it is possible the downstream bioactive inflammatory mediators were enriched. We decided to examine proinflammatory cytokines and chemokines during phagocytosis, which serve as important chemical messengers to trigger the inflammation cascade including neutrophil recruitment, edema and hemorrhaging. Both mRNA and protein levels were examined during CR-mediated phagocytosis and compared to FcγR-mediated phagocytosis and control BMDMs. CR-mediated phagocytosis provoked significant increases in cytokine transcript and secretion of the potent pyrogen TNF-α (26) in murine macrophages. Similar results were observed for IL-6, which along with TNF-α, is secreted rapidly upon macrophage stimulation to induce fever and stimulate the acute phase of the immune response (26). Additionally, the cytokine array revealed a CR-specific upregulation of the chemokine MCP-5/CCL12, which recruits eosinophils, monocytes and lymphocytes to sites of inflammation (26).

A priority for our work was resolving the mechanism behind CR-mediated proinflammatory cytokine expression. Our preliminary observations indicated that phagocytic receptors signal downstream to NF-κB, but not to AP-1. Upregulation of cytokines can be induced by stimuli that lead to the phosphorylation and ubiquitination and degradation of IκBα, which results in the nuclear translocation of active NF-κB (38). While we did not observe significantly more NF-κB in the nucleus during phagocytosis, we did see significantly reduced cytosolic sequestration of NF-κB during CR-mediated phagocytosis. Importantly, inhibition of NF-κB activation blunted proinflammatory cytokine production during CR-mediated phagocytosis. Our immunoblotting data of IκBα, indicated that both CR- and FcγR-mediated phagocytosis reduce IκBα levels in macrophages, supporting NF-κB activation during phagocytosis. Although the potential activation of NF-κB was not exclusive to CR-mediated phagocytosis, it may explain the slight increase in proinflammatory cytokines observed during FcγR-mediated phagocytosis in BMDMs. Calpain was implicated in cytokine gene induction during CR-mediated phagocytosis as inhibition of calpain significantly reduced cytokine transcripts and secreted protein levels in BMDMs ingesting C3bi-sRBCs. Future studies should directly examine whether IκBα is a substrate for calpain in macrophages and how it coordinates with the 26S proteasome in mediating IκBα degradation. Calpain inhibitors are being developed as effective anti-inflammatory drugs (48) and our results may provide mechanistic insight into their effectiveness.

During an immunological challenge, the goal of immune cells is ultimately to remove the invader with minimal self-tissue damage. The production of cytokines during an immunological challenge is likely temporally-regulated by the potency of the stimulus. LPS is a known potent stimulus of the inflammatory cascade but the potency of opsonins has remained unclear. Enhanced proinflammatory cytokine expression by macrophages ingesting complement-coated targets may help explain the early role for complement in infections to mobilize the adaptive immunity. We only observed slight, but not significant, induction of proinflammatory cytokines during FcγR-mediated phagocytosis in murine macrophages. These results could be due to the absence of FcγRIIa in mice (49). Future studies should directly compare the macrophage species to resolve these discrepancies, but in light of Fc signaling, our data may be relevant to human thrombosis patients who frequently have FcγRIIa mutations (50). The bulk of FcγR signaling and cytokine induction research has been in the context of opsonized pathogens, where co-receptor signaling is involved, and amplified, by FcγR activation (49, 51). Additionally, it is very difficult to try to fit FcγR and CR signaling into defined inflammatory categories because negative feedback mechanisms induced via inhibitory receptors can dampen the response by inducing anti-inflammatory molecules (5, 52).

While we observed significant upregulation of proinflammatory mediators during CR-mediated phagocytosis, the fold-change increases were often an order of magnitude lower than LPS stimulation. Thus, while detectable, the “danger signal” induced by opsonic receptor ligation is modest compared to signals induced by microbe-associated molecular patterns. This is to be expected as normal clearance of sRBCs is not a state of infection. We employed sRBCs and latex beads as target particles to tease out individual opsonic receptor signaling effects on cytokine induction. Additionally, these “inert” particles are relevant to phagocytosis events *in vivo*. RBC opsonization and phagocytosis are observed during senescence and in patients with beta thalassemia (53). Latex beads are composed of polystyrene, an emerging FDA-approved biomaterial (54) and serve as a model for foreign bodies known to be opsonized and inflammatory in nature (55). Thus, these studies add insight into opsonic receptor signaling and importantly reveal novel aspects regarding the under-studied CR mode of phagocytosis. Together our work indicates that opsonic receptors signal differently to the nucleus and additional differences will become apparent as other molecular players are investigated in detail.

## Data Availability Statement

All datasets generated for this study are included in the article.

## Ethics Statement

The animal study was reviewed and approved by the University of Toronto Local Animal Care Committee.

## Author Contributions

DA, XL, and RH performed the experiments, data analysis, prepared the figures, and wrote the first draft of the manuscript. REH provided feedback and guidance on all aspects of the project and wrote the final version of the manuscript.

### Conflict of Interest

The authors declare that the research was conducted in the absence of any commercial or financial relationships that could be construed as a potential conflict of interest.

## References

[1] Wynn TA Chawla A Pollard JW. Macrophage biology in development, homeostasis and disease. Nature. (2013) 496:445–55. 10.1038/nature12034 23619691 PMC3725458

[2] Naik U Harrison RE Phagocytosis. In: Nabi, editor. Building Blocks of the Cell. San Rafael, CA: Morgan & Claypool Life Sciences (2013). p. 1–118. 10.4199/C00081ED1V01Y201304BBC004

[3] Indik ZK Park JG Hunter S Schreiber AD. The molecular dissection of Fc gamma receptor mediated phagocytosis. Blood. (1995) 86:4389–99. 10.1182/blood.V86.12.4389.bloodjournal86124389 8541526

[4] Vidarsson G Dekkers G Rispens T. IgG subclasses and allotypes: from structure to effector functions. Front Immunol. (2014) 5:520. 10.3389/fimmu.2014.00520 25368619 PMC4202688

[5] Nimmerjahn F Ravetch JV. Fcgamma receptors: old friends and new family members. Immunity. (2006) 24:19–28. 10.1016/j.immuni.2005.11.010 16413920

[6] Hibbs ML Selvaraj P Carpen O Springer TA Kuster H Jouvin MH. Mechanisms for regulating expression of membrane isoforms of Fc gamma RIII (CD16). Science. (1989) 246:1608–11. 10.1126/science.2531918 2531918

[7] Bruhns P Jonsson F. Mouse and human FcR effector functions. Immunol Rev. (2015) 268:25–51. 10.1111/imr.12350 26497511

[8] Sanchez-Mejorada G Rosales C. Fcgamma receptor-mediated mitogen-activated protein kinase activation in monocytes is independent of Ras. J Biol Chem. (1998) 273:27610–9. 10.1074/jbc.273.42.27610 9765295

[9] Crowley MT Costello PS Fitzer-Attas CJ Turner M Meng F Lowell C. A critical role for Syk in signal transduction and phagocytosis mediated by Fcgamma receptors on macrophages. J Exp Med. (1997) 186:1027–39. 10.1084/jem.186.7.1027 9314552 PMC2199061

[10] Hackam DJ Rotstein OD Sjolin C Schreiber AD Trimble WS Grinstein S. v-SNARE-dependent secretion is required for phagocytosis. Proc Natl Acad Sci USA. (1998) 95:11691–6. 10.1073/pnas.95.20.11691 9751727 PMC21702

[11] Getahun A Cambier JC. Of ITIMs, ITAMs, and ITAMis: revisiting immunoglobulin Fc receptor signaling. Immunol Rev. (2015) 268:66–73. 10.1111/imr.12336 26497513 PMC4621791

[12] Bajic G Degn SE Thiel S Andersen GR. Complement activation, regulation, and molecular basis for complement-related diseases. EMBO J. (2015) 34:2735–57. 10.15252/embj.201591881 26489954 PMC4682646

[13] Wills-Karp M. Complement activation pathways: a bridge between innate and adaptive immune responses in asthma. Proc Am Thorac Soc. (2007) 4:247–51. 10.1513/pats.200704-046AW 17607007 PMC2647626

[14] Van Lookeren Campagne M Wiesmann C Brown EJ. Macrophage complement receptors and pathogen clearance. Cell Microbiol. (2007) 9:2095–102. 10.1111/j.1462-5822.2007.00981.x 17590164

[15] Tegla CA Cudrici C Patel S Trippe R III Rus V Niculescu F. Membrane attack by complement: the assembly and biology of terminal complement complexes. Immunol Res. (2011) 51:45–60. 10.1007/s12026-011-8239-5 21850539 PMC3732183

[16] Wright SD Griffin FM Jr. Activation of phagocytic cells' C3 receptors for phagocytosis. J Leukoc Biol. (1985) 38:327–39. 10.1002/jlb.38.2.327 3897421

[17] Allen LA Aderem A. Molecular definition of distinct cytoskeletal structures involved in complement- and Fc receptor-mediated phagocytosis in macrophages. J Exp Med. (1996) 184:627–37. 10.1084/jem.184.2.627 8760816 PMC2192718

[18] Tzircotis G Braga VM Caron E. RhoG is required for both FcgammaR- and CR3-mediated phagocytosis. J Cell Sci. (2011) 124:2897–902. 10.1242/jcs.084269 21878497

[19] Patel PC Harrison RE. Membrane ruffles capture C3bi-opsonized particles in activated macrophages. Mol Biol Cell. (2008) 19:4628–39. 10.1091/mbc.e08-02-0223 18768756 PMC2575151

[20] Takeuchi O Akira S. Pattern recognition receptors and inflammation. Cell. (2010) 140:805–20. 10.1016/j.cell.2010.01.022 20303872

[21] Aderem AA Wright SD Silverstein SC Cohn ZA Ligated complement receptors do not activate the arachidonic acid cascade in resident peritoneal macrophages. J Exp Med. (1985) 161:617–22. 10.1084/jem.161.3.617 3156206 PMC2187583

[22] Wright SD Silverstein SC Receptors for C3b and C3bi promote phagocytosis but not the release of toxic oxygen from human phagocytes. J Exp Med. (1983) 158:2016–23. 10.1084/jem.158.6.2016 6227677 PMC2187185

[23] Collins HL Bancroft GJ. Cytokine enhancement of complement-dependent phagocytosis by macrophages: synergy of tumor necrosis factor-alpha and granulocyte-macrophage colony-stimulating factor for phagocytosis of Cryptococcus neoformans. Eur J Immunol. (1992) 22:1447–54. 10.1002/eji.1830220617 1601035

[24] Cross CE Collins HL Bancroft GJ. CR3-dependent phagocytosis by murine macrophages: different cytokines regulate ingestion of a defined CR3 ligand and complement-opsonized Cryptococcus neoformans. Immunology. (1997) 91:289–96. 10.1046/j.1365-2567.1997.00238.x 9227330 PMC1363860

[25] Aderem A Underhill DM. Mechanisms of phagocytosis in macrophages. Annu Rev Immunol. (1999) 17:593–623. 10.1146/annurev.immunol.17.1.593 10358769

[26] Arango Duque G Descoteaux A. Macrophage cytokines: involvement in immunity and infectious diseases. Front Immunol. (2014) 5:491. 10.3389/fimmu.2014.00491 25339958 PMC4188125

[27] Wright SD Silverstein SC. Tumor-promoting phorbol esters stimulate C3b and C3b' receptor-mediated phagocytosis in cultured human monocytes. J Exp Med. (1982) 156:1149–64. 10.1084/jem.156.4.1149 7153708 PMC2186805

[28] Kang CD Han CS Kim KW Do IR Kim CM Kim SH. Activation of NF-kappaB mediates the PMA-induced differentiation of K562 cells. Cancer Lett. (1998) 132:99–106. 10.1016/S0304-3835(98)00165-7 10397459

[29] Gstraunthaler G. Alternatives to the use of fetal bovine serum: serum-free cell culture. ALTEX. (2003) 20:275–81. 14671707

[30] Chow JC Young DW Golenbock DT Christ WJ Gusovsky F. Toll-like receptor-4 mediates lipopolysaccharide-induced signal transduction. J Biol Chem. (1999) 274:10689–92. 10.1074/jbc.274.16.10689 10196138

[31] Majeed M Caveggion E Lowell CA Berton G. Role of Src kinases and Syk in Fcγ receptor-mediated phagocytosis and phagosome-lysosome fusion. J Leukoc Biol. (2001) 70:801–11. Available online at: https://jlb.onlinelibrary.wiley.com/doi/full/10.1189/jlb.70.5.801?sid=nlm%3Apubmed 11698501

[32] Naik U Nguyen QPH HarrisonRE. Binding and uptake of single and dual-opsonized targets by macrophages. J Cell Biochem. (2019) 10.1002/jcb.29043 31172552

[33] Sansone P Bromberg J. Environment, inflammation, and cancer. Curr Opin Genet Dev. (2011) 21:80–5. 10.1016/j.gde.2010.11.001 21144738

[34] Huang JH Lin CY Wu SY Chen WY Chu CL Brown GD. CR3 and Dectin-1 collaborate in macrophage cytokine response through association on lipid rafts and activation of Syk-JNK-AP-1 pathway. PLoS Pathog. (2015) 11:e1004985. 10.1371/journal.ppat.1004985 26132276 PMC4488469

[35] Karin M. The regulation of AP-1 activity by mitogen-activated protein kinases. Philos Trans R Soc Lond B Biol Sci. (1996) 351:127–34. 10.1098/rstb.1996.0008 8650258

[36] Kogut MH Genovese KJ He H. Flagellin and lipopolysaccharide stimulate the MEK-ERK signaling pathway in chicken heterophils through differential activation of the small GTPases, Ras and Rap1. Mol Immunol. (2007) 44:1729–36. 10.1016/j.molimm.2006.07.292 17045653

[37] Kawai T Akira S. Signaling to NF-kappaB by Toll-like receptors. Trends Mol Med. (2007) 13:460–9. 10.1016/j.molmed.2007.09.002 18029230

[38] Lu YC Yeh WC Ohashi PS. LPS/TLR4 signal transduction pathway. Cytokine. (2008) 42:145–51. 10.1016/j.cyto.2008.01.006 18304834

[39] Shin HM Kim MH Kim BH Jung SH Kim YS Park HJ. Inhibitory action of novel aromatic diamine compound on lipopolysaccharide-induced nuclear translocation of NF-kappaB without affecting IkappaB degradation. FEBS Lett. (2004) 571:50–4. 10.1016/j.febslet.2004.06.056 15280016

[40] Mao X Pan X Cheng T Zhang X. Therapeutic potential of the proteasome inhibitor Bortezomib on titanium particle-induced inflammation in a murine model. Inflammation. (2012) 35:905–12. 10.1007/s10753-011-9392-7 21965047

[41] Franco SJ Huttenlocher A. Regulating cell migration: calpains make the cut. J Cell Sci. (2005) 118:3829–38. 10.1242/jcs.02562 16129881

[42] Shumway SD Maki M Miyamoto S. The PEST domain of IkappaBalpha is necessary and sufficient for *in vitro* degradation by mu-calpain. J Biol Chem. (1999) 274:30874–81. 10.1074/jbc.274.43.30874 10521480

[43] Norton RL Fredericks GJ Huang Z Fay JD Hoffmann FW Hoffmann PR. Selenoprotein K regulation of palmitoylation and calpain cleavage of ASAP2 is required for efficient FcgammaR-mediated phagocytosis. J Leukoc Biol. (2017) 101:439–48. 10.1189/jlb.2A0316-156RR 27601625 PMC5235904

[44] Low KE Karunan Partha S Davies PL Campbell RL. Allosteric inhibitors of calpains: reevaluating inhibition by PD150606 and LSEAL. Biochim Biophys Acta. (2014) 1840:3367–73. 10.1016/j.bbagen.2014.08.014 25196359

[45] Kohl J. Self, non-self, and danger: a complementary view. Adv Exp Med Biol. (2006) 586:71–94. 10.1007/0-387-34134-X_6 16893066

[46] Caron E Hall A. Identification of two distinct mechanisms of phagocytosis controlled by different Rho GTPases. Science. (1998) 282:1717–21. 10.1126/science.282.5394.1717 9831565

[47] Ricciotti E Fitzgerald GA. Prostaglandins and inflammation. Arterioscler Thromb Vasc Biol. (2011) 31:986–1000. 10.1161/ATVBAHA.110.207449 21508345 PMC3081099

[48] Ji J Su L Liu Z. Critical role of calpain in inflammation. Biomed Rep. (2016) 5:647–52. 10.3892/br.2016.785 28101338 PMC5228304

[49] Vogelpoel LT Baeten DL De Jong EC Den Dunnen J. Control of cytokine production by human fc gamma receptors: implications for pathogen defense and autoimmunity. Front Immunol. (2015) 6:79. 10.3389/fimmu.2015.00079 25759693 PMC4338787

[50] Buxhofer-Ausch V Olcaydu D Gisslinger B Schalling M Frantal S Thiele J. Decanucleotide insertion polymorphism of F7 significantly influences the risk of thrombosis in patients with essential thrombocythemia. Eur J Haematol. (2014) 93:103–11. 10.1111/ejh.12307 24617727

[51] Lennartz M Drake J. Molecular mechanisms of macrophage Toll-like receptor-Fc receptor synergy. F1000Res. (2018) 7:21. 10.12688/f1000research.12679.1 29375818 PMC5760967

[52] Sutterwala FS Noel GJ Salgame P Mosser DM. Reversal of proinflammatory responses by ligating the macrophage Fcgamma receptor type I. J Exp Med. (1998) 188:217–22. 10.1084/jem.188.1.217 9653099 PMC2525554

[53] Sosale NG Rouhiparkouhi T Bradshaw AM Dimova R Lipowsky R Discher DE. Cell rigidity and shape override CD47's “self”-signaling in phagocytosis by hyperactivating myosin-II. Blood. (2015) 125:542–52. 10.1182/blood-2014-06-585299 25411427 PMC4296014

[54] El Fray M Prowans P Puskas JE Altstadt V. Biocompatibility and fatigue properties of polystyrene-polyisobutylene-polystyrene, an emerging thermoplastic elastomeric biomaterial. Biomacromolecules. (2006) 7:844–50. 10.1021/bm050971c 16529422

[55] Nilsson B Ekdahl KN Mollnes TE Lambris JD. The role of complement in biomaterial-induced inflammation. Mol Immunol. (2007) 44:82–94. 10.1016/j.molimm.2006.06.020 16905192

